# Study protocol of a randomized controlled trial for the synergizing effects of rTMS and Tui Na on upper limb motor function and cortical activity in ischemic stroke

**DOI:** 10.3389/fneur.2022.993227

**Published:** 2022-11-11

**Authors:** Yu-Feng Chen, Guang-Yue Zhu, Meng-Chai Mao, Ya Zheng, Hao Huang, Lan-Lan Liu, Si-Yun Chen, Ling-Yun Cao, Dong-Sheng Xu

**Affiliations:** ^1^School of Rehabilitation Science, Shanghai University of Traditional Chinese Medicine, Shanghai, China; ^2^Department of Tui Na, Hangzhou Dingqiao's Hospital, Hangzhou, China; ^3^Rehabilitation Medical Center, Tongji Hospital Affiliated to Tongji University School of Medicine, Shanghai, China; ^4^Engineering Research Center of Traditional Chinese Medicine Intelligent Rehabilitation, Ministry of Education, Shanghai, China; ^5^Department of Rehabilitation Medicine, Shanghai Third Rehabilitation Hospital Affiliated to Shanghai University of Traditional Chinese Medicine, Shanghai, China; ^6^Department of Rehabilitation Medicine, Yueyang Hospital of Integrated Traditional Chinese and Western Medicine, Shanghai, China

**Keywords:** repetitive transcranial magnetic stimulation (rTMS), Tui Na (Chinese massage), functional near-infrared spectroscopy (fNIRS), sensorimotor neural circuits, upper limb motor dysfunction, stroke

## Abstract

Upper limb motor dysfunction after stroke is a serious threat to the living quality of patients and their families. Recovery of upper limb motor function after stroke largely relies on the activation and remodeling of neural circuits. rTMS (repetitive transcranial magnetic stimulation) has been proved to promote the reconstruction of neural synapses and neural circuits. However, there are still a large number of patients who cannot fully recover and leave behind varying degrees of dysfunction. Considering the systemic pathology after stroke, in addition to focal brain injury, stroke can also cause extensive dysfunction of peripheral organs. The rehabilitation strategy for stroke should combine the treatment of primary brain lesions with the intervention of secondary systemic damage. The aim of this trial is to verify the efficacy of rTMS synergize with Tui Na (Chinese Massage) on upper limb motor function after ischemic stroke, and to explore the mechanism of activation and remodeling of sensorimotor neural circuits with functional near-infrared spectroscopy. Ninety patients will be randomly assigned to either rTMS + Tui Na + conventional rehabilitation group (the experimental group) or rTMS + conventional rehabilitation group (the control group) in 1:1 ratio. Intervention is conducted five sessions a week, with a total of twenty sessions. The primary outcome is Fugl-Meyer Assessment, and the secondary outcomes include Muscle Strength, Modified Ashworth Assessment, Modified Barthel Index Assessment, motor evoked potentials and functional near-infrared spectroscopy. There are four time points for the evaluation, including baseline, 2 weeks and 4 weeks after the start of treatment, and 4 weeks after the end of treatment. This study is a randomized controlled trial. This study was approved by Institutional Ethics Committee of Shanghai Third Rehabilitation Hospital Affiliated to Shanghai University of Traditional Chinese Medicine (approval No. SH3RH-2021-EC-012) on December, 16th, 2021. The protocol was registered with Chinese Clinical Trial Registry (ChiCTR2200056266), on February 3th, 2022. Patient recruitment was initiated on February 10th, 2022, and the study will be continued until December 2023.

## Background

Epidemiological investigation shows that stroke seriously threatens safety and quality of life in the world (1). Ischemic stroke accounts for a higher proportion than that of hemorrhagic stroke. Most patients have motor dysfunction, especially upper limb (UL) motor dysfunction after stroke (2). UL movement is more delicate and complex, and its unsatisfactory recovery can reduce patients' activities of daily life and seriously affect the living quality of patients and their families (3). Therefore, UL motor dysfunction is a key and challenging point in rehabilitation after stroke.

Transcranial magnetic stimulation (TMS) is a neuromodulation technique for treating nervous and mental diseases in recent years. Repetitive transcranial magnetic stimulation (rTMS) is a continuous, repetitive mode of TMS, and it has been proved that rTMS has a good effect on neurological disorders. The latest guideline in 2019 recommended TMS as an A level for treating UL motor dysfunction in subacute stroke (4). Research has shown that rTMS can promote the regulation of microenvironmental factors, glial cell activity, and nerve fiber myelin sheath repair in the injured area, and promote the reconstruction of neural synapses and neural circuits (5–7). However, there are still a large number of patients who cannot fully recover and leave behind varying degrees of dysfunction (8). Therefore, while continuously improving the technology of rTMS, such as accuracy and treatment depth, it is necessary to find other methods to synergize with rTMS to enhance the therapeutic effect. Currently, there are some deficiencies in the combination between rTMS and other treatments (such as neuromuscular electrical stimulation, physical therapy and occupational therapy). The treatment mainly focuses on the brain and limbs, but does not pay attention to the influence of systemic pathology on the recovery of UL, and there is no good intervention method for systemic pathological injury (9).

Considering the systemic pathology after stroke, in addition to focal brain injury, stroke can also cause extensive dysfunction of peripheral organs. Stroke will profoundly change the autonomic nervous system, hypothalamus-pituitary-adrenal axis, immune system and fascia and muscle system, resulting in further systemic damage (10). In turn, systemic pathological changes after stroke promote the progress and prognosis of brain injury to a great extent. Especially in the elderly, it usually leads to more serious systemic damage and a worse prognosis after stroke (9). Before the stroke, the central and peripheral organ systems of a large number of elderly patients have been affected by varying degrees of structural and functional degradation because of continuous exposure to normal aging or chronic low-grade systemic inflammation and other pathological backgrounds. All of these “silent” pathology before stroke have laid the foundation for the emergence of systemic abnormalities caused by stroke (9). After the stroke, excitotoxicity, neuroinflammation, energy disturbance, oxidative stress, and other pathological processes work together, combined with previous complications, to cause or aggravate the structural changes and functional damage of multiple peripheral organs (11). Therefore, stroke should be regarded as a systemic disease with a profound impact on the surrounding organs. The rehabilitation strategy for stroke should combine the treatment of primary brain lesions with the intervention of secondary systemic damage.

Tui Na (Chinese massage therapy) is a relatively simple, inexpensive and non-invasive intervention, and has been used to treat stroke patients for many years in China. Tui Na mainly acts on specific parts of the body through manipulation, on the one hand, it plays a local therapeutic role in the body, on the other hand, it can also have a certain impact on various systems of the body through nerves, body fluids and other ways, so as to achieve the role of treating diseases (12). Clinical observation suggested that Tui Na might be a safe and effective treatment for post-stroke UL motor dysfunction (13, 14). Although the mechanism of Tui Na on treatment of post-stroke motor dysfunction is limited, some possible mechanism was proposed. Tui Na manipulation stretched the muscle–tendon complex and stimulate the Golgi tendon organ that could inhibit alpha motor neurons and reduce spasm (15). Tui Na manipulation might reinforce sensory stimulation and activate the gamma efferent fibers of muscle stretch receptors that make receptors more sensitive to stretch (15). Soreness and distension that was caused by Tui Na manipulation will excite the sensory cortex, thus inhibit reticular formation of brainstem and decrease the muscle tone (15). For more than two thousand years, traditional Chinese medicine therapists have been using Tui Na act on governor meridian-related acupoints to treat spinal-related diseases and many visceral disorders (16). The spine is the pillar of the human body and the medium that connects the brain and the peripheral organs. The spine is the pathway of the governor meridian in traditional Chinese medicine, which dominates people's life activities (17). The spine contains the spinal cord, with spinal nerves and sympathetic pathways on both sides. Although there are great differences in theory and technology between traditional Chinese and western medicine, they are almost the same in understanding the importance of the spine and preventing and treating diseases by adjusting the spine and its related surrounding tissues.

Based on the above research background, we adopt the strategy of rTMS to synergize with Tui Na to promote the recovery of UL motor dysfunction after ischemic stroke. fNIRS (functional near-infrared spectroscopy) will be used as a technique for sensorimotor cortex activity monitoring and brain plasticity evaluation. The function of Tui Na is reflected in two aspects:1. Tui Na will be used to stimulate the peripheral sensory nerve, promote the activation of the cortical sensorimotor network, and then activate the corticospinal tract with rTMS from the cortical motor area (M1) aimed at the reconstruction of “sensory afferent-cortical integration-motor execution,” in order to obtain the effect of synergy-enhancement. 2. The extensive effects of Tui Na on autonomic nerve, fascia and muscle system can promote the function of a wide range of peripheral organs, to promote the prognosis of stroke. Therefore, our primary objective was to identify a protocol for a randomized controlled trial to evaluate the effects of rTMS synergizing with Tui Na on the motor function of the UL in patients with ischemic stroke. We hypothesized that the treatment of rTMS synergized with Tui Na can promote the improvement of UL motor function after ischemic stroke and activate and remodel sensorimotor cortex activity.

## Methods/design

**Figure 1:**
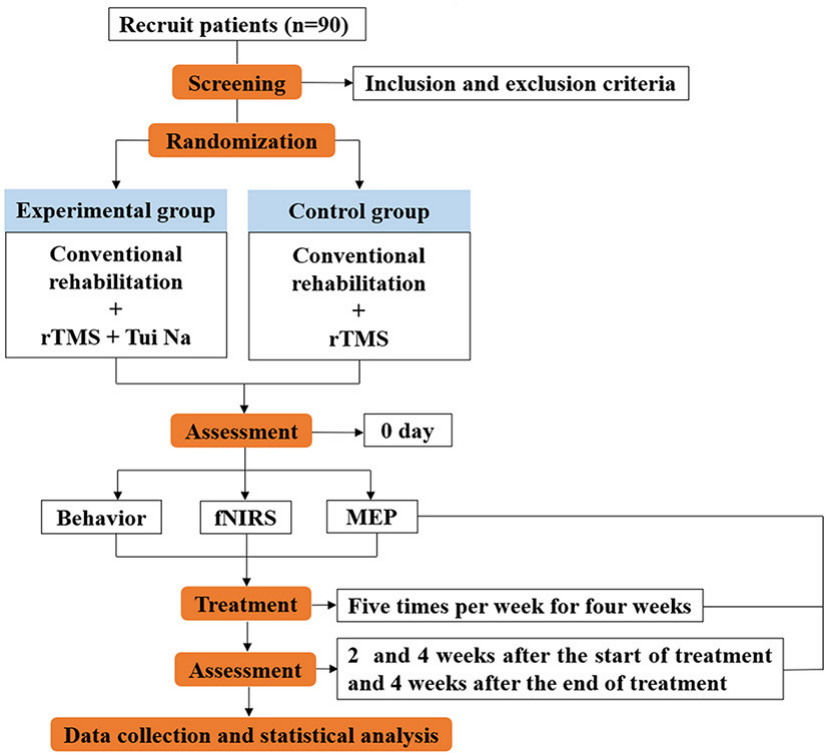
Summary of the study design.

The study is a prospective, single-center, randomized, parallel-controlled design. Ninety patients will be randomly assigned to either rTMS + Tui Na + conventional rehabilitation group (the experimental group) or rTMS + conventional rehabilitation group (the control group) in 1:1 ratio. There are four time points to evaluate the efficacy, including baseline, 2 weeks and 4 weeks after the start of treatment, and 4 weeks after the end of treatment. Intervention will be conducted five sessions a week, with a total of twenty sessions (Figure 1). Each patient will be required to sign an informed consent form before participation. Patients will be informed of the purpose of the research, the procedures involved, the expected completion time, potential adverse reactions and possible benefits. The protocol is registered with Chinese Clinical Trial Registry (ChiCTR2200056266), registered on February 3^th^, 2022.

### Eligibility criteria and recruitment

We will screen stroke patients who had treatment at Shanghai Third Rehabilitation Hospital Affiliated to Shanghai University of Traditional Chinese Medicine from February 2022 to December 2022 based on electronic medical records.

### Inclusion criteria

People will be eligible for inclusion if they meet the following criteria: (1) First ever unilateral ischemic stroke diagnosed by imaging and the course of the disease ranged from 2 weeks to 6 months. (2) UL dysfunction as indicated by the Fugl-Meyer motor function score ≥20-40/66. (3) Aged 40–70 years, regardless of sex. (4) No severe cognitive or communication impairment as indicated by the Mini-mental state examination (MMSE) ≥ 24 points.

### Exclusion criteria

Exclusion criteria are as follows: (1) Patients with severe systemic diseases such as cardiovascular or pulmonary diseases who cannot tolerate rehabilitation treatment. (2) Patients diagnosed with mental illness such as severe depression, etc. (3) In addition to stroke, suffering from other neurological diseases such as Parkinson's disease and epilepsy, etc. (4) According to the safety guidelines, there are contraindications for TMS, such as metal foreign bodies or other electronic devices implanted in the body, etc. (5) Application of drugs that change the excitability of the cerebral cortex. (6) Patients with unstable or larger carotid plaques. (7) Patients with severe osteoporosis.

### Sample size calculation

The main purpose of the experiment is to compare whether the curative effect of the experimental group is better than that of the control group. The main evaluation index of curative effect will be the change of upper limb Fugl-Meyer scale. The calculation of the sample size will be based on data from previous research and our previous experiment (18). As the study involves two groups, the sample size calculation formula for a two-sample mean comparison is as follows:


(1) $$\begin{array}{l} n_{c}=\frac{{\left(z_{1-\alpha }+z_{1-\beta }\right)}^{2}\sigma^{2}\left(1+\frac{1}{K}\right)}{{\left(\mu_{T}-\mu_{C}-\Delta \right)}^{2}} \end{array}$$

where, Z_1_-α = 1.960, Z_1_-β = 0.842, σ = 2.0 (standard deviation), K = 1(ratio of participants in the experimental and control groups), 1 = 3 (optimality bounds), α = 0.025 (2.5% one-tailed significance level), and β = 0.20 (80% power), μ_T_ = 6.6 (predicted mean in experimental group); μ_C_ = 2.3 (predicted mean in control group). The calculated required sample size is 38 participants for each group. Assuming the dropout rate is 20%, 45 participants per group (total of 90 participants) need to be recruited.

### Recruitment strategies

Qualified patients will be recruited by Shanghai Third Rehabilitation Hospital Affiliated to Shanghai University of Traditional Chinese Medicine in Shanghai. The hospital will publish research advertisements to recruit patients. Clinicians will recruit patients strictly following the inclusion and exclusion criteria set in this study. To improve recruitment quality and efficiency, investigators will monitor medical records. The investigators will carefully ask about the patient's past and current medical history, as well as the information related to the experiment, and record it in detail. Patients will be fully informed of the study details.

### Research groups

Control group: conventional rehabilitation + rTMS.

The conventional rehabilitation treatments include physiotherapy therapy, occupational therapy and drug therapy. High-frequency rTMS will be used on the affected side of the primary motor cortex (M1).

Experimental group: conventional rehabilitation + rTMS + Tui Na.

In addition to the conventional rehabilitation treatment and high-frequency rTMS provided to the patients in the control group, the patients will receive Tui Na on the spine and hemiplegic side of UL.

### Randomization and blinding

A simple randomization method will be used in this experiment. Individuals recruited in the experiment will be divided into two groups. Ninety random numbers will be generated by Excel and the random distribution table will be determined by sorting method. In this study, the envelope random allocation method will be used to put the cards printed with the allocation group into sequentially coded, opaque and sealed envelopes one by one according to the random distribution sequence. When the researchers determined that the subjects are qualified, a researcher who did not participate in the treatment, evaluation and statistics opened the envelopes sequentially and assigned the subjects to an appropriate group. An experienced blinded therapist will perform the assessment, he/she is one of the clinical therapists in the hospital. Emergency unblinding will be performed if a serious adverse event occurs. In the event of unblinding during the study, the involved subject will be removed from the study protocol. After trial completion, participant grouping will be revealed, and data analysis will commence. The research data will be properly preserved by specialized researcher. The blind data and the database will be submitted to experimental statistics professionals for statistical analysis and statistical analysis report will be written. After the statistical report is completed, the principal researcher will write a summary report of the research.

## SPIRIT diagram of enrolment, interventions and assessments

### Intervention

**Figure 2:**
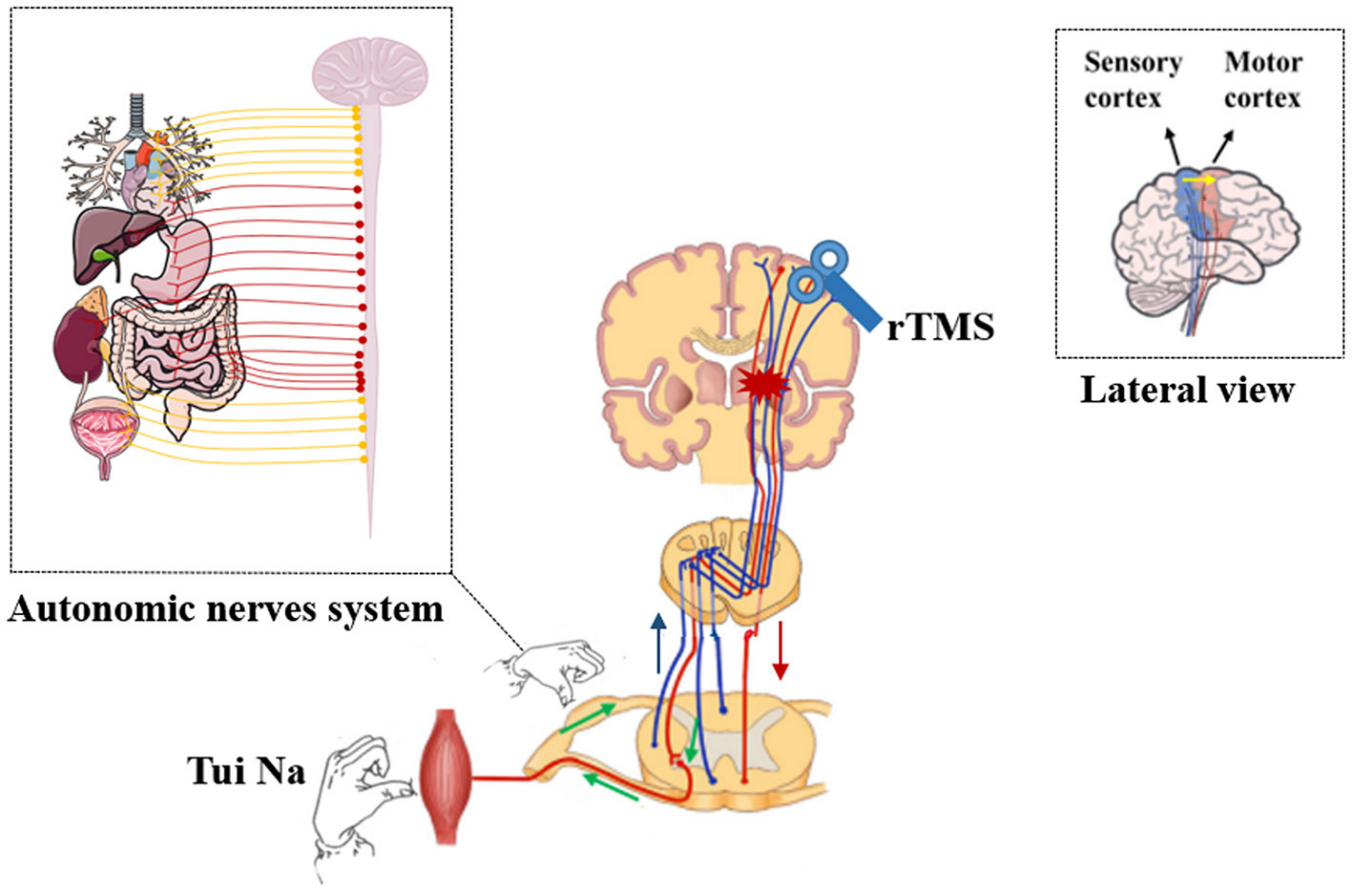
rTMS synergize with Tui Na treatment.

rTMS treatment will be performed first, and Tui Na treatment will be performed immediately after rTMS treatment. All patients will receive the treatment plan for twenty sessions over 4 weeks with Tui Na for 30 min and rTMS for 10 minevery session (Figure 2).

#### rTMS

rTMS intervention will be performed using a transcranial magnetic stimulator (MagTD; Wuhan Yiruide Medical Equipment New Technology Co., Ltd., Wuhan, China) and a figure of eight coil (YRD, maximum magnetic field intensity = 2T, diameter = 9 cm, Wuhan Yiruide Medical Equipment New Technology Co., Ltd.). First, the resting motion threshold (RMT) of rTMS will be determined. The surface recording electrode will be placed at the abdomen of the abductor pollicis brevis muscle on the affected side (if MEP is not detected, the unaffected side will be used) of the UL, the reference electrode will be placed at the tendon, and the ground wire will be connected to the wrist. Adjust the central position of the magnetic stimulation coil to the best position of motor evoked potential (MEP) of abductor pollicis brevis, and gradually reduce the stimulation. In the resting state, at least 5 of the ten stimuli with a minimum magnetic stimulation intensity of >50 μV MEP amplitude will be considered RMT on the side of the cerebral cortex. During the treatment, the patient will be in the supine position, and the motor area of the ipsilesional side of the brain (M1) will be used as the stimulation target. The “8” coil is tangent to the surface of the skull, the midpoint will be aimed at the stimulation target, the stimulation intensity is 100% RMT, the stimulation frequency is 10 Hz, each sequence is 20 pulses, the sequence interval is 8s, with a total of 1200 pulses in 10 min. The patients will be treated 5 times a week for 4 weeks.

#### Tui Na

##### Selection-of-acupoints-and-locations

Focus on the acupoints of LI4 (Large Intestine 4), PC8 (Peri Cardium 8), SJ5 (SanJiao), PC6 (Peri Cardium 6), LI10 (Large Intestine 10), LI11 (Large Intestine 11), LU5 (Lung 5), LI14 (Large Intestine 14), LI15 (Large Intestine 15), ST12 (Stomach meridian of foot Yangming 12) for the affected UL. Focus on the acupoints of DU14 (Governor meridian 14), DU4 (Governor meridian 4), GB21 (Gall BLadder 21), BL11 (Bladder 11), BL13 (Bladder 13), BL15 (Bladder 15), BL18 (Bladder 18), BL19 (Bladder 19), BL20 (Bladder 20), BL21 (Bladder 21), BL23 (Bladder 23) for the spine. Acupoints are rich in receptors, so select these acupoints of the affected UL to achieve a comprehensive stimulation of the affected UL. The acupoints around the spine correspond to the viscera of the whole body through the spinal nerve and sympathetic nerve. Stimulating these acupoints can regulate the organs and functions of the whole body.

##### Selection-of-manipulations-and-operating-rules

Kneading, pressing, one-finger meditation and active joint manipulations will be selected. The standard manipulation requires the basic technical requirements of “lasting, powerful, uniform, soft and penetration.” “Lasting” means that the manipulation can be operated continuously for a certain period without interruption to maintain the consistency of movement and strength and to ensure that the amount of stimulation to the body accumulates to a certain extent. “Powerful” means that the manipulation must have a certain strength and reach a certain level. This kind of power is not brute force or violence but a skillful force that is used according to the different people and locations of treatment. “Uniform” means that the manipulation's strength, speed and operating range should be homogeneous, rather than sometimes light and sometimes heavy, sometimes fast and sometimes slow, and sometimes large and sometimes small. “Soft” refers to gentle and rhythmic movements, not stiff, rough, or brute force. “Penetration” means that the stimulation of the manipulation can reach the deep subcutaneous layer and viscera tissue. The above aspects are closely related and complement each other. The continuous use of manipulations can gradually reduce the muscle tension of patients so that the strength of manipulation can gradually infiltrate into the deep tissue. Uniform and coordinated movements can soften the manipulations. The combination of strength and skill makes the manipulation both powerful and soft.

##### Procedure-of-treatment

The affected UL: the patients will be PLACED in a sitting position, and the therapist will focus on the acupoints from the hand to the shoulder to carry out kneading, pressing, one-finger meditation, and active joint manipulations. The appropriate force will be used to make the patient feel sore and distended, and the treatment time is about 15 min.

Spine: the patients will be placed in a prone position, and the therapist will focus on the acupoints from neck to waist to carry out kneading and pressing manipulations. Appropriate force will be used to make the patient feel sore and distended, and the treatment time is about 15 min.

##### Matters-needing-attention-in-treatment

Treatments should be performed in a quiet room. The temperature and humidity should be appropriate. The therapist's hands should be kept clean and warm. Nails should be trimmed frequently so as not to cause discomfort to the patient or even damage the patient's skin. Fully communicate with patients to eliminate their nervousness. Closely observe the reaction of patients to timely adjust the amount of stimulation, and beware of adverse responses or accidents. In the event of an accident, the treatment should be stopped immediately.

#### Conventional rehabilitation

All the patients will receive conventional rehabilitation treatment during the study period, including physical therapy, occupational therapy and drug therapy etc. The patients cannot change the conventional rehabilitation during the study period.

### fNIRS data acquisition

**Figure 3:**
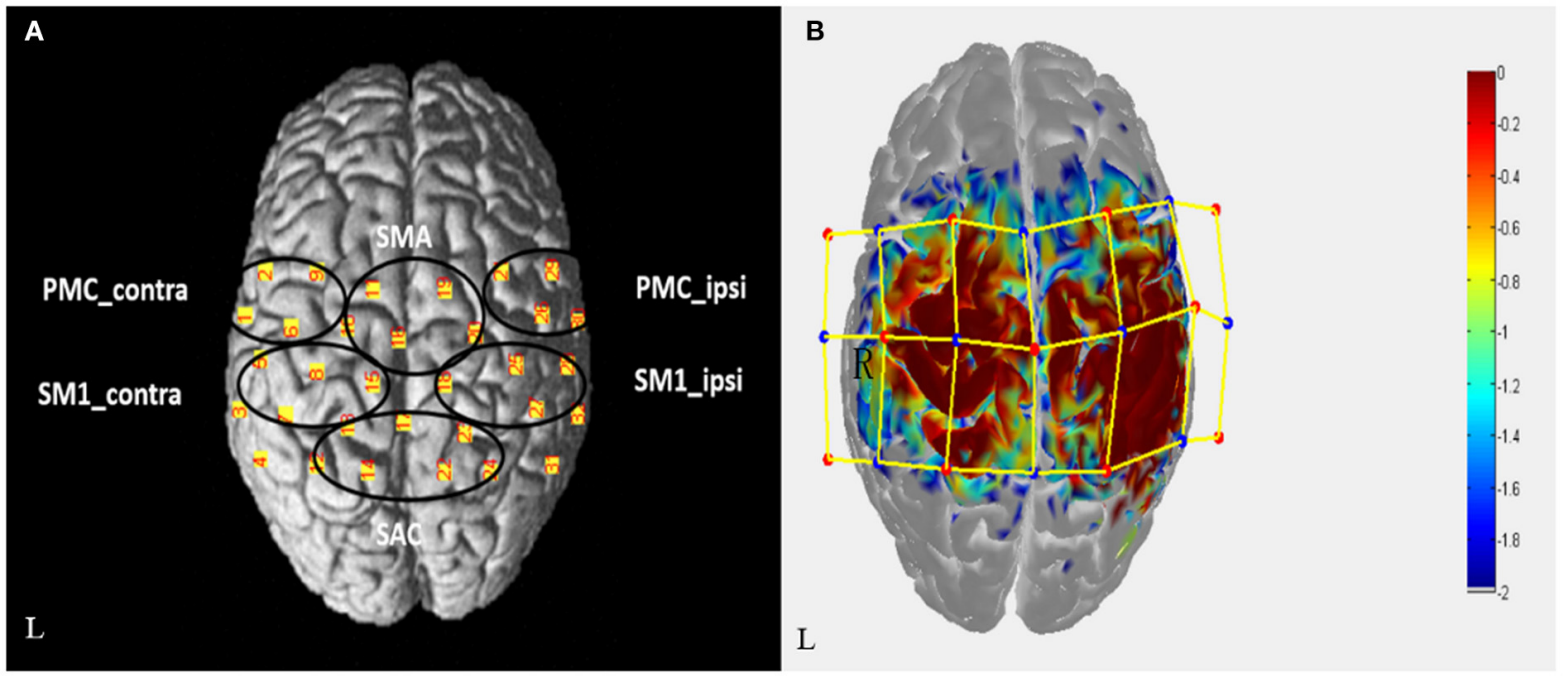
**(A)** The arrangement map of fNIRS channels in the cortex, which is divided into 6 ROIs. **(B)** Light sources (red dot, 11) and detectors (blue dot, 10) forming 32 channels (yellow line). The sensitivity distribution map of photons in the cortex is simulated by Monte Carlo, and the redder the region is, the stronger the sensitivity is.

fNIRS equipment (Danyang Huicuang Medical equipment Co., Ltd., Jiangsu, China) will be used to record the hemodynamic data of patients before and after rehabilitation treatment. A total of 21 NIRS optrodes (11 light sources and 10 detectors, with wavelength of 730 and 850 nm, and sampling rate of 19 Hz) forming 32 observation channels. The optrodes will be fixed on the patient's head with a holder, covering the bilateral frontal and parietal regions. Resting-state session and task session will be included in fNIRS evaluation. During the resting-state evaluation, patients will be asked to sit and relax for 8 min, and the resting data will be collected. The task session involved repetitive movements of flexion and extension of the hemiplegic fingers at frequency of 1 Hz with verbal cues, using a block design [six cycles: rest (10 sec); hand movements (15 sec); rest (20 sec)]. A three-dimensional digitizer (Patriot, America) will be used to collect the 3D coordinate information of the optrodes and channels to ensure the accuracy of the observed brain area and to be consistent in multiple measurements. According to the Brodmann area (BA) and anatomical locations of cortex, the observation channels will be divided into six ROIs (regions of interest): ipsilesional premotor cortex, PMC_ipsi; contralesional premotor cortex, PMC_contra; ipsilesional primary sensorimotor cortex, SM1_ipsi; contralesional primary sensorimotor cortex, SM1_contra; supplementary motor area, SMA; Somatosensory Association Cortex, SAC (Figure 3).

### fNIRS data analysis

The fNIRS data will be preprocessed and analyzed using NIRS-KIT (19) and NIRS-SPM v.3.2 (20, 21) based on MATLAB 2013b (The MathWorks Inc., Massachusetts). Considering that HbO signal has been widely used in studies of clinical interventions (22), with better sensitivity to task-related hemodynamic changes (23), and found excellent reliability for both task-related activity (24) and resting-state functional connectivity (RSFC) (25) in test-retest studies, we will focus on HbO signal in present protocol study. fNIRS data will be preprocessed with a first order detrend to remove liner trends and Temporal Derivative Distribution Repair (TDDR) algorithm to correct motion artifacts (26).

For task-related assessment, fNIRS data will be further smoothed with the canonical HRF. The General Linear Model (GLM) will be used to detect the hemodynamic activities of the NIRS time-series. The design matrix consisting of a boxcar regressor of motor task will be convolved with a Gaussian HRF to obtain the predictors of the time series of neural activation. Beta-estimates represent the weight of motor task to the variance of the hemodynamic signal of each channel. Cortical activation will then be calculated with a contrast vector of [1–1], and significance will be set at p < 0.05. All activation results will be corrected by multiple comparisons using the Tube formula method in NIRS-SPM. The number of activated channels in the contralateral and ipsilateral hemisphere will be used to calculate the Lateral Index, LI = (contralateral – ipsilateral) / (contralateral + ipsilateral).

For resting-state assessment, data segment of 7-min in the middle of resting-state session will be used to analyze RSFC. After detrend and motion-artifact correction, fNIRS data will be bandpass filtered (0.01 to 0.08 Hz) using a third-order IIR filter. RSFC will be calculated by Pearson's correlation with Fisher-z score conversion, on levels of ROI2ROI, ROI2Whole-brain, and whole brain (channel -wise). Furthermore, the amplitude of low frequency fluctuations (ALFF) will be computed by Fourier transform, and then fractional ALFF be calculated by the ratio of power spectrum of low-frequency (0.01–0.08 Hz) to that of the entire frequency range (27). The fractional ALFF values will be standardized by Fisher-Z conversion as zfALFF, for further statistics. All correlation coefficients will be first analyzed by False Discovery Rate correction (FDR correction) and then analyzed by intra-group and inter-group statistics.

### Outcome measures

The assessment will be conducted on four time points, including at baseline, 2 weeks and 4 weeks after the beginning of the treatment, and 4 weeks after the end of the treatment (Table 1). All assessments will be performed by therapists who do not know the specific group.

**Table 1:** SPIRIT figure.

	**Screening**	**Baseline**	**Intervention**			**Follow weeks**
Time Point	**-t**_**2**_	**-t**_**1**_	**0**	**w2**	**w4**	**4w**
Enrollment						
Informed consent	**√**					
Demographic information	**√**					
Medical history	**√**					
MMSE	**√**					
Eligibility assessment	**√**					
Random allocation		**√**				
Interventions						
Tui Na+TMS group						
Cnventional group						
Assessments						
Fugl-Meyer-UE		**√**		**√**	**√**	**√**
MS		**√**		**√**	**√**	**√**
MAS		**√**		**√**	**√**	**√**
MBI		**√**		**√**	**√**	**√**
MEP		**√**		**√**	**√**	**√**
fNIRS		**√**		**√**	**√**	**√**
Adverse event			**√**	**√**	**√**	**√**

Description of the protocol. -t2, first contact with participants; -t1, baseline evaluation;0, starting day of the treatment; w2, 2weeks after the start of treatment;w4, 4 weeks after the start of treatment and post evaluation;4w, 4 weeks after the end of treatment; MMSE, Mini-Mental State Examination; Fugl-Meyer-UE, Fugl-Meyer Upper Extremity Scale; MS, Muscle Strength; MAS, Modified Ashworth, MBI, Modified Barthel Index; MEP, Motor evoked potentials; fNIRS, functional near-infrared spectroscopy; The orange line represents the treatment time in the figure, with a total of 4 weeks of treatment.

### Primary outcome

#### Fugl-Meyer-UE assessment

The primary outcome measure is the Fugl-Meyer-UE Assessment (28). To evaluate the motor effects of rTMS synergized with Tui Na treatment on patients with stroke, we can also examine the changes in motor value provided by Fugl-Meyer-UE Assessment in patients. Fugl-Meyer Assessment is a method for evaluating sensorimotor disorders in stroke patients and is now widely used in the clinical evaluation of motor function. After many tests, Fugl-Meyer assessment scores have good consistency, responsiveness, and accuracy. Regarding UL motor function, the maximum Fugl-Meyer-UE score is 66, which is equivalent to the need for complete sensorimotor recovery.

### Secondary outcomes

#### Muscle strength and modified Ashworth assessment

Muscle Strength Assessment means the strength of muscle contraction during random exercise of the limb (29). The assessment is to instruct the patient to make the upper arm abduction, adduction, forearm extension and flexion, wrist extension and flexion, finger abduction, adduction, fist clenching and other movements, and observe whether the muscle strength is normal, decreased or paralyzed, and pay attention to the paralyzed site. Modified Ashworth Assessment is a method to check the tension of muscles in a state of rest and relaxation (30).

#### Modified barthel index assessment

Modified Barthel Index Assessment is a simple, reliable, sensitive and widely used method for evaluating activities of daily living. MBI is mainly used to assess the changes of independent living ability of stroke patients before and after treatment (31).

#### Motor evoked potentials

MEP is the muscle motor potential recorded by stimulating the motor cortex in the target muscle, and to examine the overall synchronization and integrity of the motor nerve transmission from the cortex to the muscle and the conduction pathway. TMS results in sequential activation of upper motor neurons, which in turn leads to spinal cord motor neuron activation, and is recorded by the surface Electromyography in the target muscle (32). In this research, at least 5 out of 10 monopulse stimuli will be given to the cortical representative region (M1) of the contralateral abductor pollicis brevis to induce MEP with an amplitude >50 μV as the motor threshold. The resting motor threshold (RMT) will be analyzed.

#### fNIRS

fNIRS will be used to record the time course of the functional plasticity of sensorimotor neural circuits during rTMS synergize with Tui Na intervention. At the time point of each evaluation, the task state and resting-state fNIRS brain imaging data of the patients will be recorded, and the cortical activity, functional connection plasticity and cortical lateralization will be calculated quantitatively (33). The fNIRS outcome measures, including Lateral Index, RSFC values and zfALFF values, will be specified in the section of fNIRS data analysis.

### Further outcome measurement

#### Adverse events

Further outcome measurements including the occurrence rate of headache, vertigo and hearing changes. Magnetic effects and strongly induced voltage during rTMS treatment may adversely affect implants in the brain or heart of patients, so patients with implants will be excluded when recruiting patients. Patients will be advised to remove magnetic objects such as necklaces, earrings and glasses during treatment to avoid safety incidents. Side effects such as headache, vertigo and hearing changes may occur during the treatment of rTMS. Once the above symptoms occur, the treatment will be stopped immediately to ensure safety. rTMS may induce epilepsy, the probability is very small, once it occurs, it will reduce the damage through the formulation of emergency treatment plan. In the process of Tui Na treatment, bone correction and other possible risk factors are not involved, and there is no serious risk.

### Statistical analysis

#### Data set population determination

In this research, the Intention to treat (ITT) and Per-Protocol set (PPS) will be used together. In line with the selection and exclusion criteria in the program, good compliance, no other related treatment will be carried out during the research, and the case report forms (CRF) table will be completed, which has an evaluable curative effect index. The patients with poor curative effect will be also included in the PPS set as ineffective cases. For missing data, we will use LOCF method to make up. We will use the Statistical Package for Social Sciences (SPSS-IBM^®^,Armonk, NY: IBM Corp.) version 20 for Windows to complete statistical analysis of this study.

### Statistical analysis plan

#### Case distribution analysis

The shedding and elimination cases will be described, and the drop rate and elimination rate of the two groups will be compared.

#### Comparability analysis

According to the analysis of the basic situation of the two groups before treatment, the sex of the patient will be divided into two variables, and the chi-square test of two independent sample rates will be used. W-test and F-test will be used to determine that the continuous variables such as Fugl-Meyer-UE, MS, MBI and MEP and fNIRS measurements (Lateral Index, RSFC values, zfALFF values) in the baseline is in accordance with normal distribution and homogeneity of variance, and two independent sample *t*-tests will be used for comparison. If these data do not conform to normal distribution or homogeneity of variance, Wilcoxon rank sum test will be used to compare the measurement data. If the comparison of the variables in the baseline between the two groups is statistically significant and professional, the variable will be analyzed as a covariable multiple efficacy index.

#### Efficacy analysis

The data in accordance with normal distribution and homogeneity of variance in the main and secondary indicators will be compared with *t*-test to see if the difference between the two groups before and after treatment is statistically significant. The rank data with non-normal distribution or uneven variance will be analyzed by Wilcoxon rank sum test.

#### Safety analysis

Adverse events and adverse reactions will be analyzed by statistical description method, and chi-square test or exact probability method will be used to compare the incidence of adverse events and adverse reactions between the two groups.

### Data management

#### Original data acquisition scheme and data input

The original data will be collected directly by paper version of ICF and evaluation equipment. Each ICF will be inputted into the database by two independent data entry personnel, and the data collected by the evaluation equipment will be classified by two independent data entry personnel. If there is any inconsistency, it will be necessary to find out the reason and correct it.

#### Data verification

According to the requirements of the plan, the data administrator verifies the data in the database, including time verification, logical verification, selection and exclusion condition verification, evaluation result verification etc. According to the verification results, in view of the questions existing in the case report form, the data manager will list the question-and-answer table and sent an inquiry to the researcher through the inspector. The researcher should answer the question as soon as possible and inform the data manager in writing. The data manager can modify, confirm and input the data according to the researcher's answer, and can issue the question-and-answer form again if necessary.

#### Data security

The database is backed up after audit, and the data will be locked by the main researchers, statisticians and ombudsmen to ensure the security of the data. Thereafter, any changes to the database can only be made with the consent of all three parties.

### Withdrawals

A participant will be withdrawn from the study, because of occurrence of severe adverse events and patient's own factors, for instance having a cerebrovascular accident, seizure, or the subject could not be contacted, or the subject is not willing to continue to join.

### Monitoring

There is an independent data monitoring committee (DMC), it will be composed of two rehabilitation researchers and one statistician who are responsible for research quality supervision and safety supervision. This research will strictly abide by the scientific research management system, and strengthen the supervision of the progress, data quality and ethical safety of the project.

### Ethical considerations

The study will be conducted in strict accordance with the relevant ethical principles in the Helsinki Declaration. The study ethics and protocol have been reviewed and approved by the ethics committee of Shanghai Third Rehabilitation Hospital Affiliated to Shanghai University of Traditional Chinese Medicine.

### Informed consent forms and privacy protection of subjects

Informed consent is the process of providing people with information to help decide whether to participate in the research as subjects. After fully understanding the research information, subjects and researchers will sign the informed consent form. The subjects can withdraw their informed consent at any time during the research, and the quality of medical care will not be adversely affected, so as to protect the rights and obligations of the subjects. During the study procedures, only the investigators and inspectors have access to the personal information of subjects, and this must occur under the strict supervision. No investigators can reveal any data associated with this clinical trial, except upon an official request by the national administration agency.

### Public dissemination and literature publication

After consensus of results and conclusions in this clinical trial are obtained, a paper describing this study will be written by professional writers in the study. The researchers will report the results truthfully even if the final results and conclusions are not desirable.

## Discussion

According to the holism of traditional Chinese medicine, the human body is an organic whole, and the various components of the human body are inseparable in structure, coordinate and complement each other in function, and influence each other pathologically. Therefore, we adopt a holistic and local approach in Tui Na therapy, which not only regulates the local rehabilitation of the UL, but also pays attention to the overall condition of the patients. Similar to acupuncture (34), Tui Na on the hemiplegic UL can stimulate the peripheral nerves, and promote the activation and integration of the cortical sensorimotor network. Similar to physical therapy (35), Tui Na on the hemiplegic UL can coordinate the balance of muscle tension between muscle groups, inhibit and control spasm, and then establish a normal movement pattern. Tui Na on the spine region can promote the adjustment of the overall function of the body. All organs in the human body are stimulated by nerves. Tui Na can regulate the function of these organs by stimulating the autonomic nervous system and improve the pathological injury of the whole body after stroke, so as to provide a better internal environment for the recovery of brain and limbs (36).

In recent years, the development of Functional magnetic resonance imaging (fMRI), electroencephalography (EEG) and fNIRS has given impetus to the research of rehabilitation. Among them, fNIRS is an emerging neuroimaging technique that indirectly reflects the intensity of neural activity by detecting the changes in blood oxygen activity in the cerebral cortex (33). fNIRS has a good temporal resolution (sampling rate up to dozens of Hz) and spatial positioning ability (centimeter scale), as well as portability, economy and good ecological validity (37). In a review published in Nature in 2008, various brain imaging techniques were compared, and fNIRS was evaluated as “an interesting compromise that involves inexpensive and portable imaging techniques” in real-time brain imaging (38). These characteristics make fNIRS very suitable for clinical practice in stroke rehabilitation evaluation and rehabilitation intervention.

The innovation of this study is mainly reflected in two aspects: one is the innovation of clinical treatment. The synergistic enhancement therapy of rTMS and Tui Na from “peripheral-central” multi-mode treatment can promote the rehabilitation of UL motor function in stroke patients and promote the combination and promotion of neuromodulation and Tui Na. The other is theoretical innovation, connect traditional Chinese medicine theory holistic view, address both symptoms and root causes with modern brain science, based on the activation and remodeling of the sensorimotor circuits, the multi-dimensional sensory stimulation of Tui Na is synergized with the rTMS which acts directly on the motor cortex. This protocol intends to provide an evidence-based proof for rTMS synergize with Tui Na on UL motor dysfunction after ischemic stroke and reveal the underlying mechanism of plastic changes in sensorimotor neural circuits.

## Ethics statement

The studies involving human participants were reviewed and approved by medical Ethics Committee of Shanghai third rehabilitation hospital affiliated to Shanghai University of traditional Chinese medicine. The patients/participants provided their written informed consent to participate in this study. Written informed consent was obtained from the individual(s) for the publication of any potentially identifiable images or data included in this article.

## Author contributions

Y-FC and D-SX are responsible for the study design. D-SX conceived the study protocol. Y-FC and S-YC are responsible for applying for ethical review. L-LL sought ethical approval. Y-FC and L-YC wrote the manuscript. G-YZ and M-CM contributed to the statistical analyses. G-YZ and YZ contributed to the development of the study database. HH and L-LL is responsible for clinical recruitment. All authors carefully reviewed, agree to final script, contributed to writing the manuscript for the protocol, and approved the final version of the manuscript.

## Funding

The research was supported by National Key Research and Development Program of China (2020YFC2004202) and National Natural Science Foundation of China (General Program), Nos. 81974358 (both to D-SX).

## Conflict of interest

The authors declare that the research was conducted in the absence of any commercial or financial relationships that could be construed as a potential conflict of interest.

## Publisher's note

All claims expressed in this article are solely those of the authors and do not necessarily represent those of their affiliated organizations, or those of the publisher, the editors and the reviewers. Any product that may be evaluated in this article, or claim that may be made by its manufacturer, is not guaranteed or endorsed by the publisher.

## Abbreviations

TMS: transcranial magnetic stimulation
Tui Na: Chinese Massage
UL: upper limb
fMRI: functional magnetic resonance
fNIRS: functional near-infrared spectroscopy
MMSE: Mini Mental State examination
RMT: resting motion threshold
MEP: motor evoked potential
MS: Muscle Strength Assessment
MAS: Modified Ashworth Assessment
MBI: Modified Barthel Index Assessment
PPS: Per-Protocal set.
